# Compartmentalized into Bacteriocytes but Highly Invasive: the Puzzling Case of the Co-Obligate Symbiont Serratia symbiotica in the Aphid *Periphyllus lyropictus*

**DOI:** 10.1128/spectrum.00457-22

**Published:** 2022-06-01

**Authors:** François Renoz, Mélanie Ribeiro Lopes, Karen Gaget, Gabrielle Duport, Marie-Christine Eloy, Benoît Geelhand de Merxem, Thierry Hance, Federica Calevro

**Affiliations:** a Biodiversity Research Centre, Earth and Life Institute, UCLouvain, Louvain-la-Neuve, Belgium; b Université de Lyon, INSA Lyon, INRAE, BF2I, UMR203, Villeurbanne, France; c Louvain Institute of Biomolecular Science and Technology, UCLouvain, Louvain-la-Neuve, Belgium; University of Vienna

**Keywords:** aphids, bacterial mutualism, bacteriocytes, co-obligate *Serratia symbiotica*, embryo invasion, gut symbiont

## Abstract

Dependence on multiple nutritional symbionts that form a metabolic unit has evolved many times in insects. Although it has been postulated that host dependence on these metabolically interconnected symbionts is sustained by their high degree of anatomical integration (these symbionts are often housed in distinct symbiotic cells, the bacteriocytes, assembled into a common symbiotic organ, the bacteriome), the developmental aspects of such multipartner systems have received little attention. Aphids of the subfamilies Chaitophorinae and Lachninae typically harbor disymbiotic systems in which the metabolic capabilities of the ancient obligate symbiont Buchnera aphidicola are complemented by those of a more recently acquired nutritional symbiont, often belonging to the species Serratia symbiotica. Here, we used microscopy approaches to finely characterize the tissue tropism and infection dynamics of the disymbiotic system formed by B. aphidicola and S. symbiotica in the Norway maple aphid Periphyllus lyropictus (Chaitophorinae). Our observations show that, in this aphid, the co-obligate symbiont *S. symbiotica* exhibits a dual lifestyle: intracellular by being housed in large syncytial bacteriocytes embedded between *B. aphidicola*-containing bacteriocytes in a well-organized compartmentalization pattern, and extracellular by massively invading the digestive tract and other tissues during embryogenesis. This is the first reported case of an obligate aphid symbiont that is internalized in bacteriocytes but simultaneously adopts an extracellular lifestyle. This unusual infection pattern for an obligate insect symbiont suggests that some bacteriocyte-associated obligate symbionts, despite their integration into a cooperative partnership, still exhibit invasive behavior and escape strict compartmentalization in bacteriocytes.

**IMPORTANCE** Multipartner nutritional endosymbioses have evolved many times in insects. In Chaitophorinae aphids, the eroded metabolic capabilities of the ancient obligate symbiont *B. aphidicola* are complemented by those of more recently acquired symbionts. Here, we report the atypical case of the co-obligate *S. symbiotica* symbiont associated with *P. lyropictus*. This bacterium is compartmentalized into bacteriocytes nested into the ones harboring the more ancient symbiont *B. aphidicola*, reflecting metabolic convergences between the two symbionts. At the same time, *S. symbiotica* exhibits highly invasive behavior by colonizing various host tissues, including the digestive tract during embryogenesis. The discovery of this unusual phenotype for a co-obligate symbiont reveals a new face of multipartner nutritional endosymbiosis in insects. In particular, it shows that co-obligate symbionts can retain highly invasive traits and suggests that host dependence on these bacterial partners may evolve prior to their strict compartmentalization into specialized host structures.

## INTRODUCTION

In many sap-feeding insects, bacterial mutualism for nutrition is based on the coexistence, within the same host, of multiple obligate bacteria that collectively function as a metabolic unit to produce nutrients that are lacking in the host’s diet (1–8). Previous observations indicate that the metabolic convergence of the bacterial cosymbionts composing these multisymbiotic systems goes hand in hand with the compartmentalization of those bacterial partners into symbiotic host cells (the bacteriocytes) that can be organized in a common symbiotic organ (the bacteriome) (1, 5–16). In aphids (Hemiptera: Aphididae), nutritional disymbiotic systems have evolved on numerous occasions in species of the subfamilies Lachninae and Chaitophorinae (4, 6, 9, 17–19). Such symbioses involve the participation of a more recently acquired obligate symbiont, often belonging to the species Serratia symbiotica, in the production of essential amino acids and vitamins that the ancestral symbiont *Buchnera aphidicola* can no longer supply alone. It has been reported that these so-called co-obligate S. symbiotica symbionts may be hosted within secondary bacteriocytes located in the immediate vicinity of the primary bacteriocytes that harbor *Buchnera* (9), a spatial organization that would ensure metabolic coordination of the two complementary symbionts according to the metabolic needs of the host (15). However, the architecture of these symbiotic systems, as well as their development, has never been investigated in detail.

In the present study, we used fluorescence *in situ* hybridization (FISH) and microscopy to examine the tissue tropism and infection dynamics from embryogenesis to the adult stage of the co-obligate symbiont *S. symbiotica* associated with the Norway maple aphid Periphyllus lyropictus (Chaitophorinae) that metabolically complements B. aphidicola for the production of tryptophan and riboflavin (17, 20, 21). Our observations show that, in this aphid, the co-obligate symbiont is compartmentalized within bacteriocytes while exhibiting highly invasive behavior, a singular trait for an aphid symbiont engaged in a co-obligatory lifestyle. Our approach highlights the importance of addressing the developmental aspects of insect-bacteria symbioses to better comprehend their diversity and evolution.

## RESULTS AND DISCUSSION

**FIG 1:**
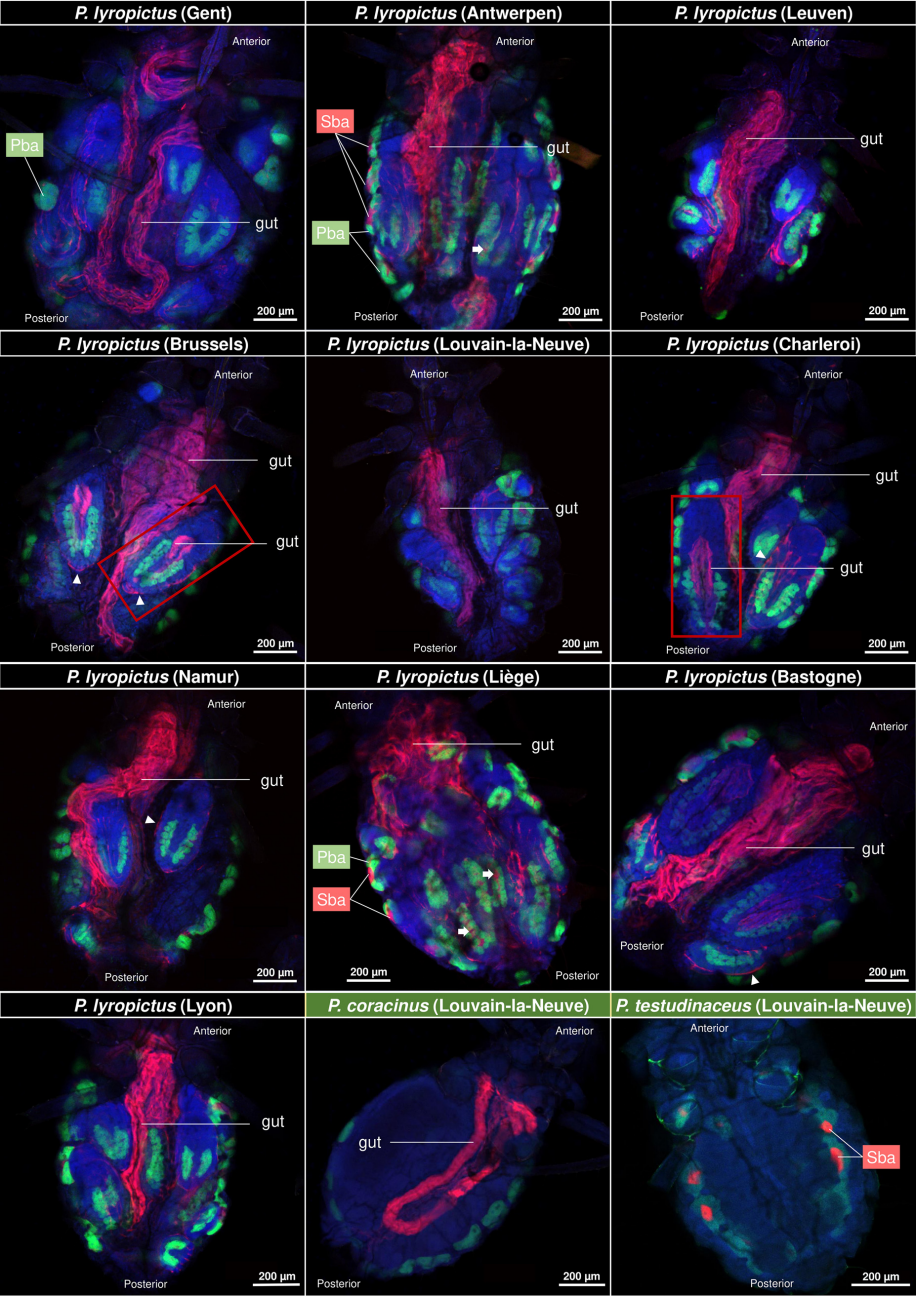
Tissue tropism of *S. symbiotica* in field-collected adult *P. lyropictus* aphids. Green, red, and blue signals indicate *Buchnera* cells, *Serratia* cells, and host insect nuclei, respectively. *S. symbiotica* was consistently observed in the gut of *P. lyropictus* adults for all 10 sites examined. *S. symbiotica* was also found in the gut of certain *P. lyropictus* embryos (see embryos surrounded by a red frame) and in secondary bacteriocytes (Sba) nested between primary bacteriocytes (Pba) harboring *Buchnera*. White arrows indicate secondary bacteriocytes in the embryos. *S. symbiotica* is also visible at the periphery of the embryos (arrowheads). *S. symbiotica* was also observed in the gut of *P. coracinus*, while it is present only in secondary bacteriocytes in *P. testudinaceus*.

To determine the general infection pattern of *S. symbiotica* associated with P. lyropictus, colonies were collected from Norway maples (Acer platanoides) during two successive sampling campaigns (2020 and 2021) throughout 9 sites in Belgium and one in Lyon (France) (Fig. S1). Colonies of other *Periphyllus* species were also sampled to compare the tissue tropism of *S. symbiotica* between species. Species identity was confirmed by c oxidase subunit I (COI) barcoding, and *B. aphidicola* and *S. symbiotica* were localized using whole-mount FISH. Our observations show that, in addition to residing in secondary bacteriocytes, *S. symbiotica* in *P. lyropictus* is also present in the digestive tract. This surprising infection pattern for a co-obligate symbiont was consistently observed in all *P. lyropictus* individuals examined (Fig. 1). *S. symbiotica* was also found in the digestive tract of Periphyllus coracinus but not in that of Periphyllus testudinaceus, where the symbiont appears to be present only in bacteriocytes (Fig. 1) (22). Furthermore, observations based on these wide shots show that *S. symbiotica* colonizes other host tissues such as the hemolymph, the periphery of embryos, and the digestive tract of late embryos.

**FIG 2:**
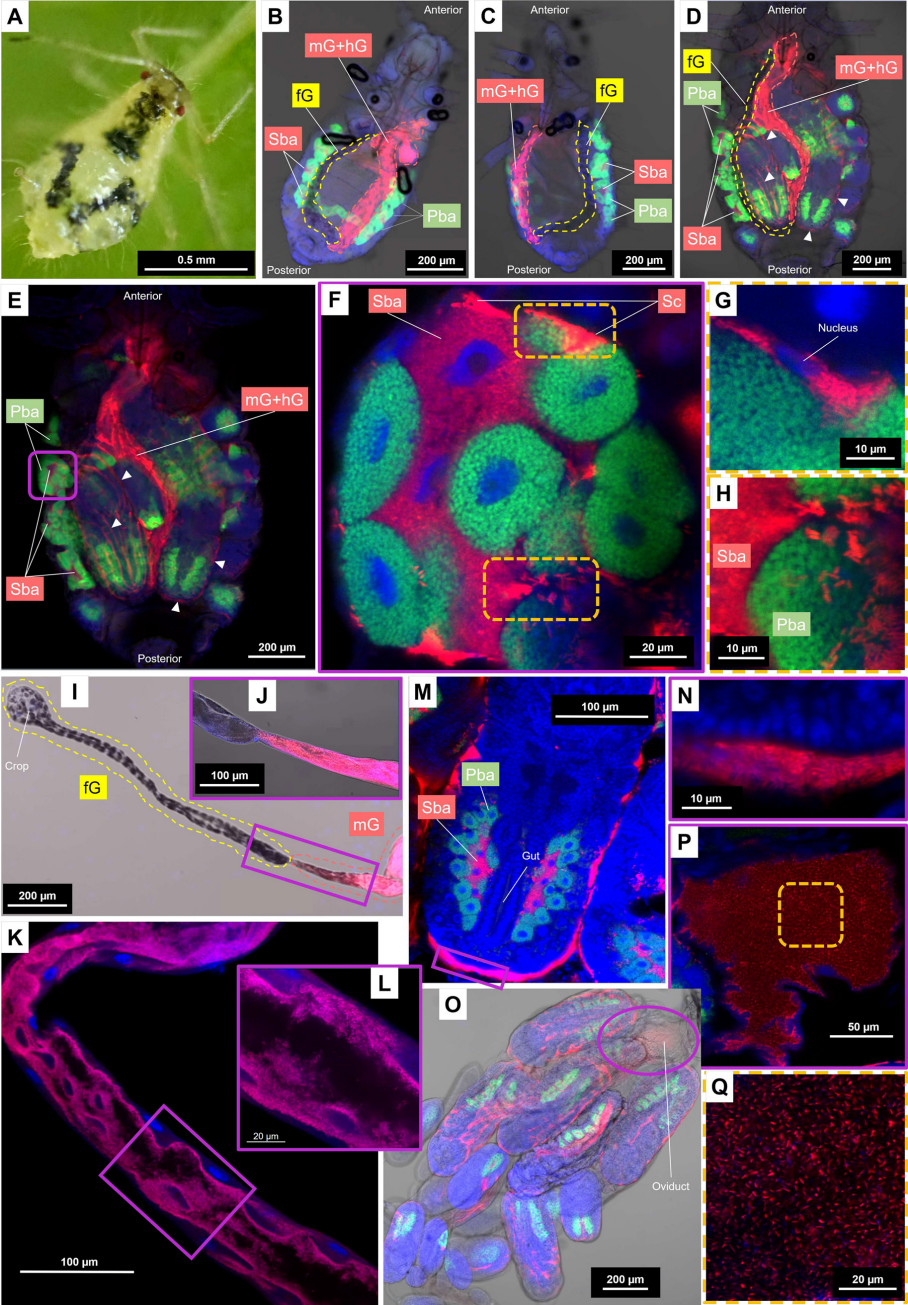
Detailed tissue tropism of the co-obligate *S. symbiotica* symbiont associated with *P. lyropictus*. Green, red, and blue signals indicate *Buchnera* cells, *Serratia* cells, and host insect nuclei, respectively. (A) A young adult of *P. lyropictus* feeding on *Acer platanoides*. (B) A second-stage nymph (3 days old; bright field) in which the horseshoe-shaped architecture of the bacteriome is well visible. *S. symbiotica* is present in secondary bacteriocytes and in the posterior part of the digestive tract (midgut and hindgut) but appears to be absent from the foregut. (C) A third instar nymph (5 days old; bright field) where *S. symbiotica* exhibits the same pattern of tissue tropism as that observed in the second instar. (D) A young adult (12 days old; bright field) where the developing embryos are more visible. *S. symbiotica* can be found in the secondary bacteriocytes, in the posterior part of the digestive tract, but also at the periphery of the embryos (arrowheads). (E) An enlarged dark field view of the adult stage from which the enlarged shots of the bacteriocyte structures that follow are derived. (F) One of the bacteriocyte clusters forming the bacteriome (Airyscan) composed of syncytial secondary bacteriocytes hosting *S. symbiotica*, embedded between the uninucleated primary bacteriocytes hosting *Buchnera*. Sheath cells that also house the co-obligate symbiont sparsely cover the periphery of the bacteriocytes. (G) Close-up view (Airyscan) of a sheath cell hosting *S. symbiotica.* (H) Close-up view (Airyscan) suggesting the occurrence of symbionts trafficking between secondary bacteriocytes and hemolymph. (I) Dissected digestive tract (bright field) demonstrating the absence of *S. symbiotica* in the foregut. (J) Close-up view (bright field) of the intersection between the foregut and the midgut confirming the presence *S. symbiotica* in the latter. (K) Midgut segment (Airyscan) showing *S. symbiotica* densely colonizing this part of the digestive tract. (L) Close-up view (Airyscan) showing *S. symbiotica* densely distributed at the inner periphery of the midgut. (M) An embryo of developmental stage 18 (Airyscan) where *S. symbiotica* is well visible in secondary bacteriocytes but also in the periphery of the embryos. (N) Close-up view (Airyscan) showing the presence of *S. symbiotica* around the embryos. (O) Dissected embryonic chain (bright field) revealing the presence of *S. symbiotica* the presence of *S. symbiotica* in the oviduct and around the embryos in the ovarioles. (P) Close-up view (Airyscan) confirming the presence of *S. symbiotica* in the oviduct. (Q) *S. symbiotica* cells (Airyscan) with their typical rod shape in the oviduct. fG, foregut; mG, midgut; hG, hindgut; Sba, secondary bacteriocyte; Pba, primary bacteriocyte; Sc, sheath cell.

**FIG 3:**
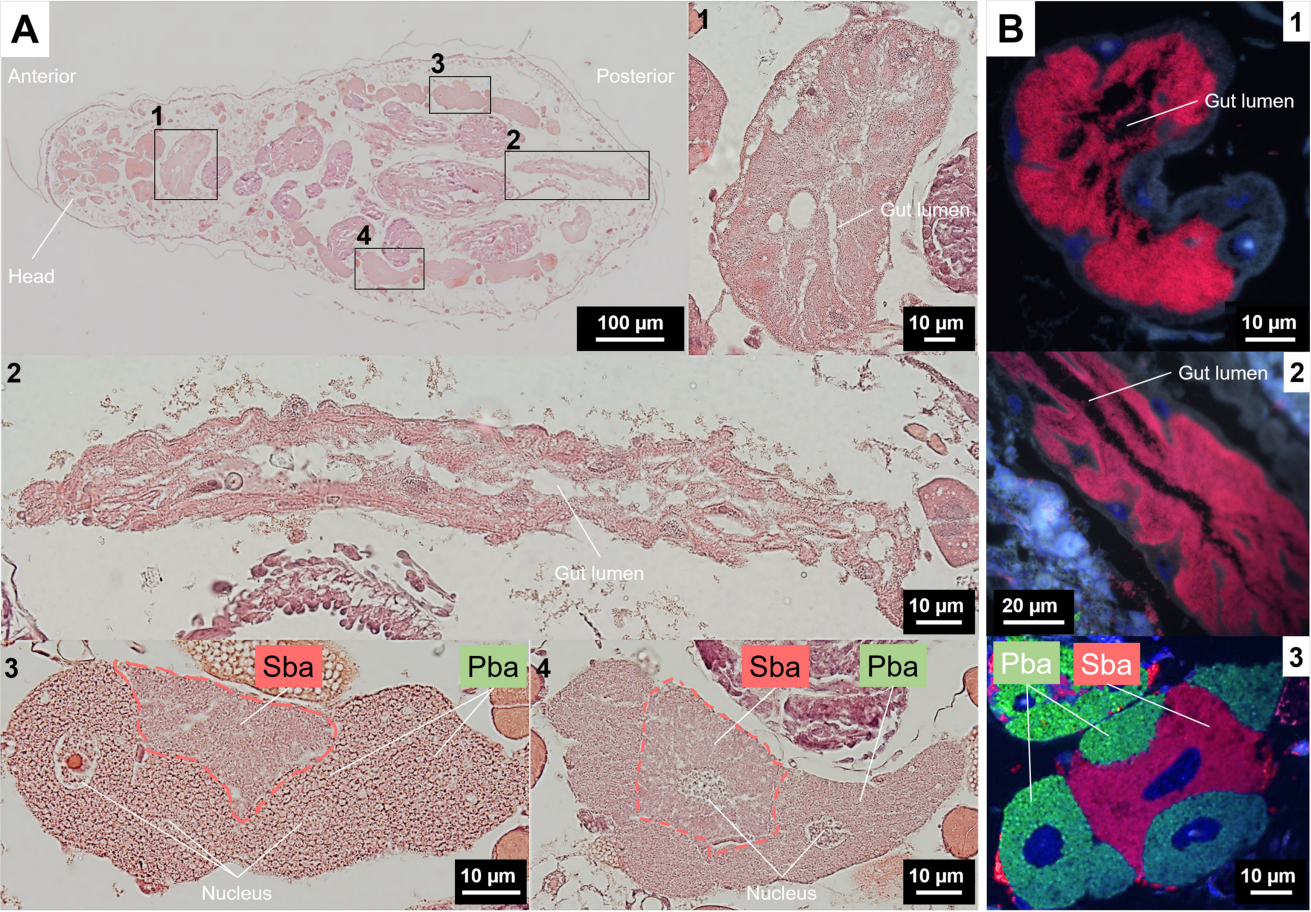
Bacteriocyte structures and tissue tropism of *S. symbiotica* in *P. lyropictus* (third instar nymph). (A) Representative images of H&E-stained whole-aphid sections. (1 and 2) Enlarged sections of the digestive tract. (3 and 4) Magnified images of H&E-stained bacteriocyte clusters showing the nested pattern of secondary bacteriocytes between primary bacteriocytes. (B) FISH on tissue sections. Green, red, and blue signals indicate *Buchnera* cells, *Serratia* cells and host insect nuclei, respectively. (1 and 2) Enlarged sections of the digestive tract showing the dense presence of *S. symbiotica*. (3) A bacteriocyte cluster consisting of primary bacteriocytes wrapping a secondary bacteriocyte. Pba, primary bacteriocyte; Sba, secondary bacteriocyte.

**FIG 4:**
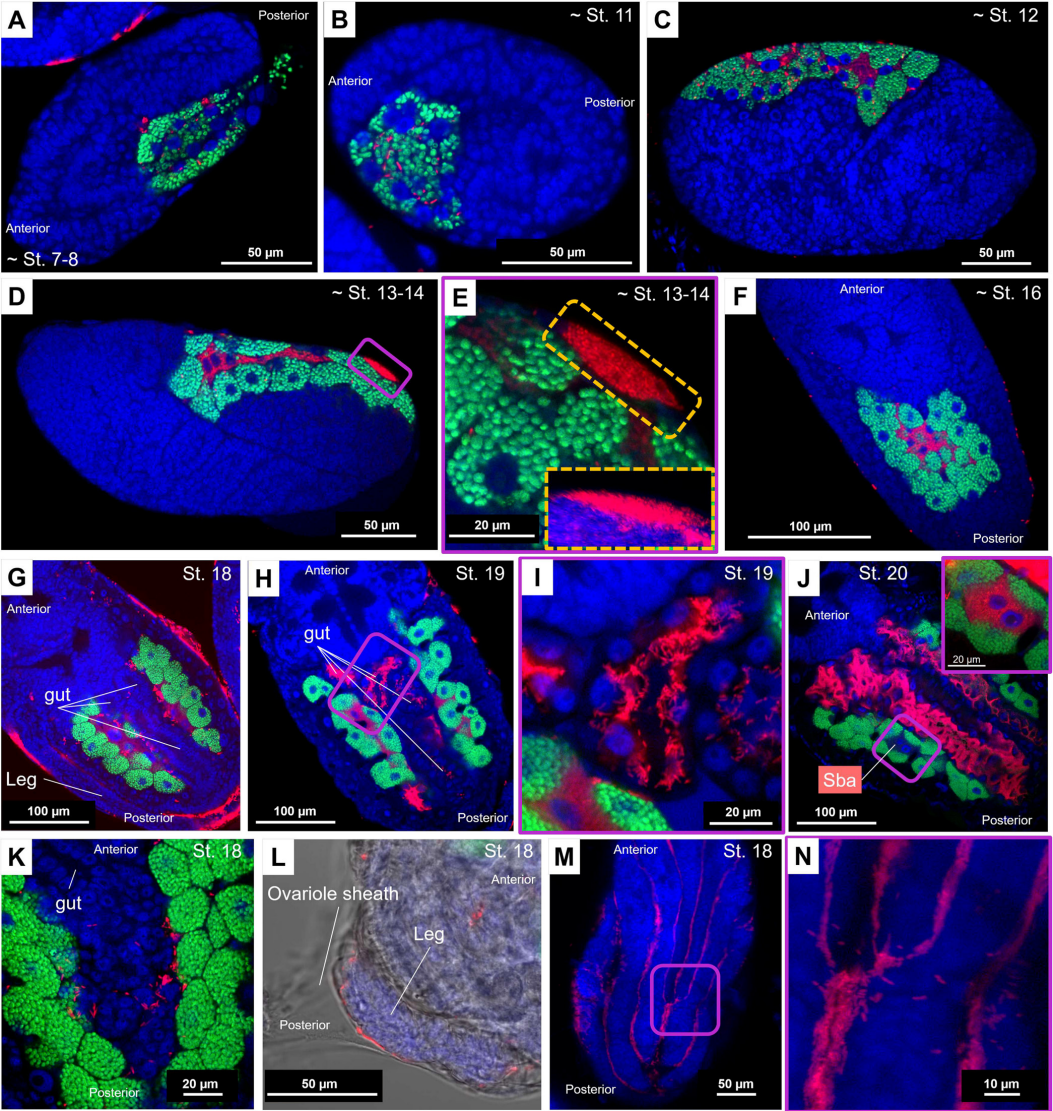
Infection dynamics of *Buchnera* and *S. symbiotica* during embryonic development in *P. lyropictus*. Green, red, and blue signals indicate *Buchnera* cells, *Serratia* cells, and host insect nuclei, respectively. (A) *Buchnera* and *S. symbiotica* infect a stage 7 to 8 embryo by transiting via the posterior zone. (B) Cellularization symbiont-infected syncytium until the achievement of stage 11. (C) Individualization of the uninucleate bacteriocytes harboring *Buchnera* during stage 12. (D) Completion of the syncytial bacteriocytes harboring *S. symbiotica*. The nuclei of the syncytial bacteriocytes are clearly visible around stages 13 to 14. (E) Local accumulation of *S. symbiotica* on the embryo surface during stages 13 to 14. Inset: z-stack sections confirming the presence of *S. symbiotica* on the surface of the embryo where they form a dense bacterial mass. (F) Establishment of a compact bacteriome in which the bacteriocytes sheltering *S. symbiotica* are surrounded by those containing *Buchnera*. *S. symbiotica* remains sporadically visible on the surface of the embryo. (G) Establishment of the horseshoe-shaped architecture of the bacteriome at stage 18. The periphery of the embryo is densely colonized by the symbiont which can even be seen between the legs. (H and I) Beginning of the colonization of the digestive tract by *S. symbiotica* at stage 19. (J) Stage 20 is marked by a massive colonization of the digestive tract by the symbiont. Inset: zoom on a secondary bacteriocyte. The fluorescent signal associated with *S. symbiotica* is so intense in the digestive tract that the visibility of the symbiont in secondary bacteriocytes is impeded. This difference in signal intensity suggests that the gut is colonized much more densely by the symbiont than by the bacteriocytes. (K) An image showing *S. symbiotica* freely circulating in the hemolymph. (L) A bright-field image showing *S. symbiotica* in the ovariole. (M) The symbiont is established in the surface invaginations of the embryos. (N) Zoom-in on these invaginations showing the concentration of the symbiont in these cavities and suggesting that *S. symbiotica* could form biofilms on the surface of the embryos.

To further investigate the tissue tropism of the co-obligate symbiont, a clone of *P. lyropictus* collected in Louvain-la-Neuve (clone LLN; Fig. 2A) was reared in the laboratory. Localization of *S. symbiotica* in both bacteriocytes and gut was first confirmed for larval and adult stages (Fig. 2B to E and Fig. 3) using FISH and microscopy techniques. In the early nymphal stages, the bacteriome forms a cohesive-looking organ with a horseshoe-shaped architecture. (Fig. 2B and C). It is composed of large syncytial bacteriocytes hosting *S. symbiotica*, embedded between the uninucleated primary bacteriocytes hosting *B. aphidicola* (Fig. 2F). During nymphal development, the horseshoe shape tends to fade, revealing more distinct clusters in the abdomen, located near the developing embryos (Fig. 2D to E and Fig. 3). Despite these morphological changes, the secondary bacteriocytes appear always embedded between the primary bacteriocytes in embryonic, nymphal, and adult stages. Facultative *S. symbiotica* symbionts have been previously reported in the so-called secondary bacteriocytes of the pea aphid Acyrthosiphon pisum (20, 23, 24), but in that case, they are less densely present than those observed in *P. lyropictus* and their distribution among the primary bacteriocytes is more disordered. The nested architecture of the *P. lyropictus* bacteriome, which resembles that of the Diaphorina citri psyllid bacteriome where the peripheral layer of unicellular bacteriocytes that contain the obligate symbiont Carsonella ruddii surrounds a central multinucleate syncytium that contains the co-obligate symbiont Profftella armatura (25), reflects the metabolic convergence between *S. symbiotica* and *B. aphidicola*. However, unlike the co-obligate symbionts reported so far in other sap-feeding insects (15), including the Lachninae aphids (9), the co-obligate *S. symbiotica* associated with *P. lyropictus* is not only confined to bacteriocytes: it is also present in sheath cells that sparsely cover the periphery of the bacteriocytes (Fig. 2G) and extracellularly, with a dense bacterial trafficking taking place between the hemolymph and the bacteriocytes (Fig. 2H). Until now, such tissue tropism had been reported only for facultative symbionts (26). Another unusual feature for a bacteriocyte-associated obligate symbiont is its ability to infect the host’s digestive tract, a feature previously reported only for pathogenic strains of *S. symbiotica* (20, 27). Regarding the infection at this anatomical level, bright-field pictures of both nymphs and adults show that only a portion of the digestive tract is infected by *S. symbiotica* (Fig. 2B to D). This is confirmed by observations of the dissected adult digestive tract, which clearly show that the foregut is not infected (Fig. 2I and J), whereas the co-obligate symbiont massively colonizes the inner periphery of the midgut and hindgut (Fig. 2K and L and Fig. S2). This is an important issue because it suggests that the populations of *S. symbiotica* residing in the gut are not the result of oral acquisition from the external environment as has been reported for other insect groups (e.g., stink bugs can acquire obligate symbionts through this route) (28). The scenario of an acquisition from the external environment is also ruled out by the presence of the symbiont in the digestive tract of late embryos (Fig. 1 and Fig. 4H to J), which suggests that the infection comes from the *S. symbiotica* bacteria already present in maternal tissues. We did not observe in our samples the large bacteriome inhabiting *S. symbiotica* described by Monnin et al. (17), and, based on our analyses, we propose that the organ reported by these authors is in fact the invaginated loop of the digestive tract that is massively infected by *S. symbiotica*. Finally, our observations also show that *S. symbiotica* colonizes the periphery of the embryos (Fig. 2M and N) and the oviduct (Fig. 2O to Q).

Such remarkable versatility in terms of tissue tropism for a co-obligate aphid symbiont raises the question of how infections take place in each tissue type and specifically during embryonic development. To address this question, embryos were dissected and processed by whole-mount FISH. Overall, the co-obligate *S. symbiotica* symbiont in *P. lyropictus* exhibits infection dynamics similar to those of facultative intracellular strains associated with the pea aphid Acyrthosiphon pisum (23) (for a detailed description of the key steps, see Fig. 4A to G) but with some major differences and notable features for an obligate aphid symbiont. The most striking difference from facultative strains is the colonization of the posterior part of the *P. lyropictus* digestive tract by *S. symbiotica* at the end of embryogenesis (i.e., stages 19 to 20) (Fig. 4H to J). Prior to these stages, the symbiont is absent from this compartment (Fig. 4G), demonstrating that its infection occurs after internalization of *S. symbiotica* into bacteriocytes. Interestingly, the symbiont is densely present in the hemolymph, around the bacteriocytes (Fig. 4K) in a fashion similar to that observed in adults (Fig. 2H), which is an uncommon infection pattern for obligate aphid symbionts that had been reported to be present exclusively intracellularly in bacteriocytes and occasionally in sheath cells (6, 9). The third major observation, which has not been noted for facultative strains in A. pisum, is that embryogenesis is marked by the colonization of the surface of the embryos (Fig. 4F to H). *S. symbiotica* massively colonizes the periphery of embryos in ovarioles (Fig. 2M to O and Fig. 4L), especially in surface invaginations (Fig. 4M and N). We hypothesize that this infection arises from local accumulation of symbionts on the surface of embryos that occurs at stages 13 to 14 (Fig. 4D and E) and extends into later developmental stages. This is a critical point because it means that this co-obligate symbiont multiplies not only inside the embryo but also outside and thus is featured by a phenotype more invasive than that of the previously reported facultative *S. symbiotica* symbionts.

Taken together, all these observations emphasize the propensity of the co-obligate *S. symbiotica* symbiont of *P. lyropictus* to adopt both an intracellular and extracellular lifestyle. They reveal a new infection pattern for an obligate aphid symbiont and more broadly for a bacteriocyte-associated obligate symbiont of sap-feeding insect (1, 5–10). In light of these observations, two scenarios could explain the origin of the infection in the gut. The first is that populations of *S. symbiotica* dwelling in the hemolymph and/or bacteriocytes are the source of the gut infection. This scenario is supported by recent observations showing that injection of culturable pathogenic *S. symbiotica* strains into the hemolymph of adult pea aphids leads to an infection of the digestive tract of the offspring (20). The second hypothesis is that the populations residing in the gut derive from those multiplying on the surface of developing embryos and in the oviducts; the proximity of the vagina and anus may facilitate the migration of the symbiont from the reproductive system to the digestive tract where it would find environmental conditions in the hindgut and midgut conducive to rapid multiplication.

Compartmentalization of symbiotic bacteria into specialized host cells or organs is a key step in establishing interdependent nutritional cooperation in insect-microbial symbioses, in which confinement in bacteriocytes ensures the control of symbiont populations by the host and the coordination of metabolic exchanges according to its development (29, 30). At the same time, adopting such an intimate lifestyle with the host promotes strong selection for avirulence, resulting in the loss of genes associated with infectivity and thus a decrease in the ability of these bacteria to colonize a wider range of niches (20, 31). The *S. symbiotica* symbiont associated with *P. lyropictus* represents a fascinating case of co-obligate symbiont, associated with an unexpected phenotype in the context of the evolution of bacterial mutualism in insects. Indeed, it is more invasive than previously reported cases of facultative aphid symbionts despite its obligate nature (23, 24, 32, 33) and escapes strict compartmentalization into host-specific structures. The *S. symbiotica* symbiont associated with *P. lyropictus* has a genome size of 2.58 Mb, the largest genome described so far for a co-obligate *S. symbiotica* symbiont (17). Genome analysis of this symbiont revealed that many virulence factors, including a large set of flagellum-related genes, are conserved compared to the genomes of other co-obligate *S. symbiotica* symbionts (21). Although the actual functionality of these factors remains unknown, it is tempting to propose that they may contribute to the propensity of this *S. symbiotica* symbiont to colonize such a wide range of tissues (e.g., a complete flagellum would facilitate the mobility of the symbiont and, therefore, its ability to invade various tissue types). Further studies are needed to determine whether the ability of this obligate symbiont to avoid strict compartmentalization into bacteriocytes is due to its capacity to further escape host control, e.g., through a functional flagellar system, quorum sensing, and biofilm formation once colonization sites are reached. In this scenario, the association between *S. symbiotica* and *P. lyropictus* would represent a case of obligate symbiosis that is not yet fully stabilized, most likely because *S. symbiotica* is at a very early stage of its establishment as a vertically transmitted obligate symbiont. In this context, the scenario of an ongoing replacement of an older strain by a younger one cannot be excluded (9, 34). Another hypothesis to consider is that in *P. lyropictus*, *S. symbiotica* colonization of the midgut and hindgut is related to specific yet unknown functions. In other insect groups, such as stink bugs, associated nutritional symbionts colonize the digestive tract, but they are sheltered in specialized invaginations of the organ and sometimes in flanking bacteriocytes (35–38). However, close examination of the midgut and hindgut of *P. lyropictus* suggests that they lack similar structures and that *S. symbiotica* massively colonizes the inner surface of these intestinal regions in an apparently unstructured manner (Fig. 4I to L and Fig. S2).

It has previously been asserted that the *S. symbiotica* symbiont associated with *P. lyropictus* was a co-obligate associate strictly confined within symbiotic cells and that this anatomical integration reflected the antiquity of the association and a high degree of metabolic interconnection between this symbiont and *B. aphidicola* (17). However, by showing that this co-obligate symbiont escapes strict compartmentalization into bacteriocytes and exhibits highly invasive traits, our study suggests instead that it is a *S. symbiotica* symbiont that has only recently become an obligate nutritional partner. This also suggests that metabolic dependence on co-obligate symbionts may evolve prior to their strict compartmentalization into specialized host structures and that the anatomical integration (i.e., compartmentalization into specialized cells and or organs) of nutritional obligate symbionts may be not as predictable as stated previously (17, 39), at least in Chaitophorinae aphids that have established nutritional disymbiotic systems involving *S. symbiotica* as a co-obligate partner. Compared to nutritional symbionts that are strictly confined in bacteriocytes, the “open lifestyle” of these co-obligate symbionts may give them more opportunities to exchange genetic material with other bacteria, which could lead them on very different evolutionary paths.

Interestingly, the unusual tissue tropism reported here for the *S. symbiotica* symbiont of *P. lyropictus* may not be an isolated case in *Periphyllus* aphids. Indeed, P. coracinus also appears to harbor an invasive *S. symbiotica* symbiont, while P. testudinaceus appears to harbor an *S. symbiotica* symbiont residing only in bacteriocytes. The results obtained in the latter cases should be taken with caution because the prevalence of *S. symbiotica* in these species, as well as the tissue tropism and infection dynamics of the symbionts, has not been studied in detail as in *P. lyropictus*. Furthermore, there are no publicly available genomic sequences for the symbionts associated with these aphids and thus no insight into their biological meaning. Nevertheless, it is likely that these differences in tissue tropism found in *Periphyllus* aphids can be explained by different evolutionary histories between *S. symbiotica* and its host species and are related to levels of anatomical integration that reflect the coevolutionary processes specific to each association. Future studies should address this hypothesis by combining genomic data with detailed analyses of symbiont tissue tropism.

In conclusion, by reporting the anatomical organization of an aphid disymbiotic nutritional system and the unusual invasive behavior of a co-obligate symbiont, our study provides new insights into the evolutionary developmental biology of bacterial mutualism in insects. The developmental bases of insect multisymbiotic systems have received little attention, and a central question that future studies should address is how metabolically complementary symbionts coordinate with and are controlled by the host. The mechanisms underlying this coordination are likely to vary depending on the degree of anatomical integration of the symbionts in the host, i.e., whether the symbionts are compartmentalized into symbiotic cells (such as bacteriocytes and sheath cells) or capable of invading other types of tissue. *Periphyllus* aphids, and more broadly aphid species of the Chaitophorinae subfamily, are likely suitable candidates to investigate these complex issues.

## MATERIALS AND METHODS

### Insect sample collection.

Colonies of the aphid species *Periphyllus lyropictus* (Hemiptera: Aphididae, Chaitophorinae) were collected on Norway maples (*Acer platanoides*) during the spring and summer 2020 and 2021. Nine sites distributed throughout Belgium were examined, as well as one site in France (city of Lyon) (Fig. S1). One colony was examined for each site. The aphid collection consisted of the entire colony, i.e., wingless parthenogenetic adult females and nymphs that, were kept in acetone until use. In addition, colonies of other *Periphyllus* species feeding on Acer campestre and Acer pseudoplatanus were collected for comparative analysis.

### Aphid identification.

Aphid DNA extraction was performed on a pool of one to three aphids from each colony using a salt-precipitation method (40). Primers LepF and LepR (5′-ATTCAACCAATCATAAAGATATTGG-3′ and 5′-TAAACTTCTGGATGTCCAAAAAATCA-3′) were used to amplify the target 658-bp fragment of cytochrome c oxidase subunit I (COI) gene (41, 42). The PCR assays were performed in a final volume of 15 μL, containing 1 μL of genomic DNA, 0.5 μM each primer, 200 μM dNTPs, 1× buffer, and 0.625 units of *Taq* DNA polymerase (Roche). The thermocycling profile was as follows: 94°C for 1 min; 6 cycles of 94°C for 1 min, 45°C for 1 min and 30 s, and 72°C for 1 min and 15 s, followed by 36 cycles of 94°C for 1 min, 51°C for 1 min and 30 s, and 72°C for 1 min and 15 s, with a final 5-min extension period of 72°C. Amplicons were purified before sequencing (Microsynth AG, Balgach, Switzerland). Insects were then identified into species by comparing resulting COI sequence data to GenBank nucleotide database using BLAST.

### Aphid clonal lines and rearing.

A colony of *P. lyropictus* collected in Louvain-la-Neuve in spring 2020 was reared in the laboratory on young Norway maples (A. platanoides) at 20°C with a photoperiod of 16 h light, 8 h dark. From this colony, one individual was used to establish a clonal line named “LLN.” This clonal line was used for dissections (bacteriocytes, digestive tracts, and embryos) and the detailed study of the tissue tropism of S. symbiotica.

### Whole-mount fluorescence *in situ* hybridization.

**(i) Protocol on whole aphids.** To establish the general tissue tropism of *S. symbiotica* and *B. aphidicola* in whole *Periphyllus* aphids, whole-mount fluorescence *in situ* hybridization (FISH) was performed as described previously (43). Insects preserved in acetone were placed in 70% ethanol for 30 min to soften the tissues. They were then transferred to Carnoy solution and, after dissection of the legs, left at room temperature overnight for fixation. After a thorough washing with 100% ethanol, the aphid samples were bleached in alcoholic 6% H_2_O_2_ solution and incubated at room temperature until they were decolorized. The bleached samples were successfully washed with 100% ethanol and 70% ethanol and then hydrated with 0.3% Triton X-100 in phosphate-buffered saline with Tween 20 (PBSTx) containing 0.3% Triton X-100 and finally incubated with hybridization buffer (20 mM Tris-HCl [pH 8.0], 0.9 M NaCl, 0.01% SDS, 30% formamide) containing 100 nM (each) probes and 0.5 μM SYTOX Green (Thermo Fisher Scientific) overnight. The following oligonucleotide probes were used for *in situ* hybridization: Cy5-PeriBuch (5′-Cy5-CCTTTTTTGGGCAGATTC-3′) targeting 16S rRNA of B. aphidicola (17) and Cy3-PASSisR (5′-Cy3-CCCGACTTTATCGCTGGC-3′) targeting 16S rRNA of *S. symbiotica* (44). To specifically localize *B. aphidicola* in *Periphyllus testudinaceus*, the probe Cy5-ApisP2a (5′-Cy5-CCTCTTTTGGGTAGATCC-3′) was used (44). After this incubation period, the samples were washed thoroughly with PBSTx, mounted in SlowFade antifade solution (Thermo Fisher Scientific), and observed under a Zeiss LSM 710 confocal microscope equipped with Airyscan detector. Observations were conducted on at least 10 individuals per colony for aphids collected in the field, as well as for individuals corresponding to each stage of the LLN clone maintained in the laboratory.

**(ii) Protocol for dissected tissues.** To perform *in situ* hybridization on dissected digestive tracts and embryos, the procedure followed was practically the same as the one performed on whole aphids with the following differences: a dissection directly performed in 70% ethanol and a bleaching period reduced to 3 to 4 days. Observations on the dissected digestive tracts were made on 15 adults taken at random from the LLN clone maintained in the laboratory. Regarding the embryos, observations were carried out on the same clone during seven different observation sessions with about 10 embryonic chains each time (each embryonic chain contained between 20 and 30 embryos at different stages, and two embryonic chains are present in each aphid). We adopted the developmental staging reported by Miura et al. (45) and Koga et al. (23).

### Hemalum/eosin staining and *in situ* hybridization on tissue sections.

To further investigate the structure of *P. lyropictus*-associated bacteriomes, sections of 10 whole *P. lyropictus* third instar nymphs were prepared and subsequently stained through previously described hemalum/eosin and *in situ* hybridization protocols (46). Briefly, aphids, with legs and antennae previously removed, were fixed by immersion in PBS with 4% paraformaldehyde and 1% Triton X-100 at 4°C for 24 h and then transferred in PBS with 4% paraformaldehyde. After several washes in PBS, samples were embedded in 1.3% agar to facilitate manipulation and then dehydrated through a series of ascending ethanol solutions, from 70% to absolute ethanol, before being transferred to 1-butanol at 4°C for 24 h. Aphids were then embedded in Paraplast (McCormick Scientific LLC, saint Louis, MO, USA) and sectioned at 3-μm thickness using an LKB Historange microtome (LKB Instruments, Bromma, Sweden). Sections were placed on polylysine-coated slides.

For histological staining, sections were deparaffinated in methylcyclohexane and rehydrated through an ethanol series to PBS. Hematoxylin and eosin (H&E) staining was performed using RAL products (RAL Diagnostics, Martillac, France) as described previously (47). After a final dehydration through a series of ascending ethanol solutions from 70% to absolute ethanol, sections were washed in Diasolv (Diapath, Martinengo, Italy) and mounted with Diamount solution (Diapath, Martinengo, Italy).

For *in situ* hybridization, deparaffinated sections were permeabilized in 70% acetic acid at 60°C for 1 min, dehydrated through a series of ascending ethanol solutions, and air-dried. Deproteinization was performed in hydrochloride acid (0.01 M) with pepsin (0.1 mg/mL) at 37°C for 10 min and again dehydrated through a series of ascending ethanol solutions. Prehybridization was then performed at 45°C for 30 min in prehybridization buffer (79% hybridization buffer [0.9 M NaCl, 20 mM Tris, 5 mM EDTA {pH 7.2}], 20% Denhardt’s solution [5 g Ficoll, 5 g polyvinylpyrrolidone, 5 g bovine serum albumin {BSA}, in 500 mL of water], and 1% SDS solution [SDS 10%]). *In situ* hybridization was performed by coating the slides at 45°C for 3 h in the dark with the oligonucleotides described above. The sections were mounted in PermaFluor aqueous mounting medium (Thermo Fisher Scientific) with DAPI (4′,6-diamidino-2-phenylindole; 3 μg/mL; Vector Laboratories). In both cases, observations were performed using an Olympus IX81 microscope (Olympus Corporation) with 10× or 60× lens magnification, and pictures were taken using an Olympus Came dia C-5060 camera (Olympus Corporation) connected to the CellSens software (Olympus Corporation).
