# Supplementary material for: Compartmentalized into Bacteriocytes but Highly Invasive: the Puzzling Case of the Co-Obligate Symbiont Serratia symbiotica in the Aphid Periphyllus lyropictus
Source: Microbiol Spectr. 2022 Jun 1;10(3):e00457-22. doi: 10.1128/spectrum.00457-22 (PMC9241954; doi:10.1128/spectrum.00457-22)

Supplemental Material for

Compartmentalized into bacteriocytes, but highly invasive: the puzzling case of the co-obligate symbiont *Serratia symbiotica* in the aphid *Periphyllus lyropictus*

François Renoz[a,b], Mélanie Ribeiro Lopes[b], Karen Gaget[c], Gabrielle Duport[c], Marie-Christine Eloy[d], Benoît Geelhand de Merxem[a], Thierry Hance[a], Federica Calevro[c]

[a]Biodiversity Research Centre, Earth and Life Institute, UCLouvain, Croix du Sud 4-5, 1348, Louvain-la-Neuve, Belgium

[b]Univ Lyon, INSA Lyon, INRAE, BF2I, UMR203, F-69621, Villeurbanne, France

[c]Univ Lyon, INRAE, INSA Lyon, BF2I, UMR203, F-69621, Villeurbanne, France

[d]Louvain Institute of Biomolecular Science and Technology, UCLouvain, Croix du sud 4-5, 1348, Louvain-la-Neuve, Belgium

Fig. S1. Summary of collection details for aphid samples.

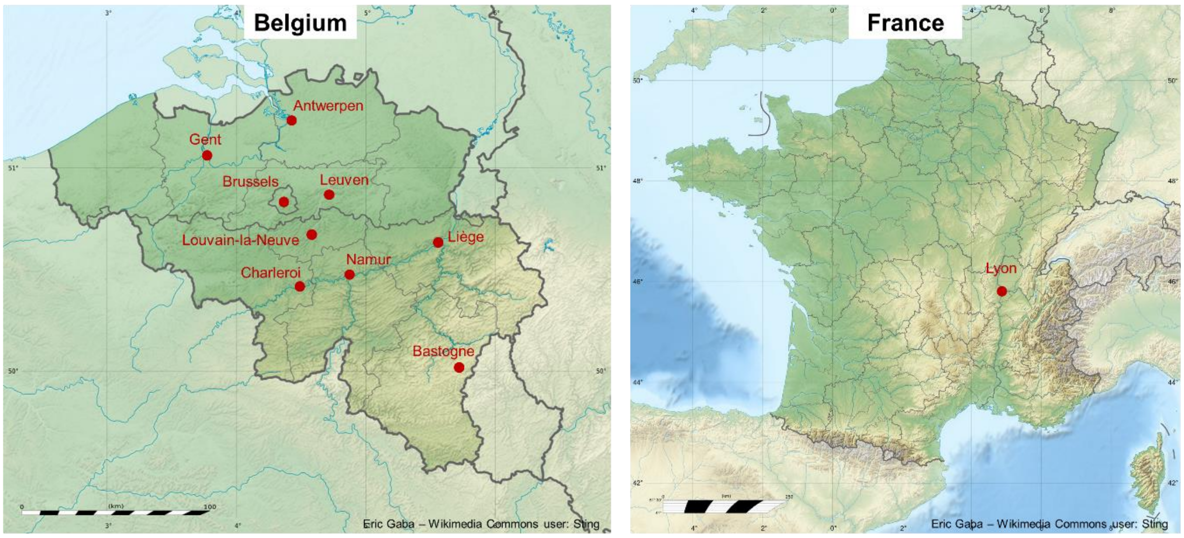

Colony no.	Aphid species	Host plant	Location	Geographical coordinates	Date
**1**	*P. lyropictus*	*Acer platanoides*	Gent	51.034168, 3.719677	29/07/2021
**2**	*P. lyropictus*	*Acer platanoides*	Antwerpen	51.185459, 4.422336	28/07/2021
**3**	*P. lyropictus*	*Acer platanoides*	Leuven	50.888564, 4.709809	02/07/2020
**4**	*P. lyropictus*	*Acer platanoides*	Brussels	50.829700, 4.429853	14/06/2020
**5**	*P. lyropictus*	*Acer platanoides*	Louvain-la-Neuve	50.666827, 4.623177	26/05/2020
**6**	*P. lyropictus*	*Acer platanoides*	Charleroi	50.408288, 4.440332	01/08/2021
**7**	*P. lyropictus*	*Acer platanoides*	Namur	50.459852, 4.861551	13/06/2020
**8**	*P. lyropictus*	*Acer platanoides*	Liège	50.608996, 5.513517	04/08/2021
**9**	*P. lyropictus*	*Acer platanoides*	Bastogne	50.001998, 5.721043	18/06/2020
**10**	*P. lyropictus*	*Acer platanoides*	Lyon	45.774230, 4.854441	14/07/2021
**11**	*P. testudinaceus*	*Acer pseudoplatanus*	Louvain-la-Neuve	50.666827, 4.623177	26/05/2020
**12**	*P. coracinus*	*Acer campestre*	Louvain-la-Neuve	50.666827, 4.623177	26/05/2020

Fig. S2. Tissue tropism of *S. symbiotica* in the digestive tract of *P. lyropictus* (adult stage). Green, red and blue signals indicate *Buchnera* cells, *Serratia* cells and host insect nuclei, respectively. (A) Whole-mount FISH of a tissue section of the foregut (Airyscan) showing the absence of *S. symbiotica* in this portion of the digestive tract. (B) Whole-mount FISH of a tissue section of the midgut (Airyscan) showing *S. symbiotica* densely colonizing the inner surface of this portion of the digestive tract. (C) Whole-mount FISH of a tissue section of the hindgut (Airyscan) showing *S. symbiotica* colonizing the inner surface of this portion of the digestive tract.

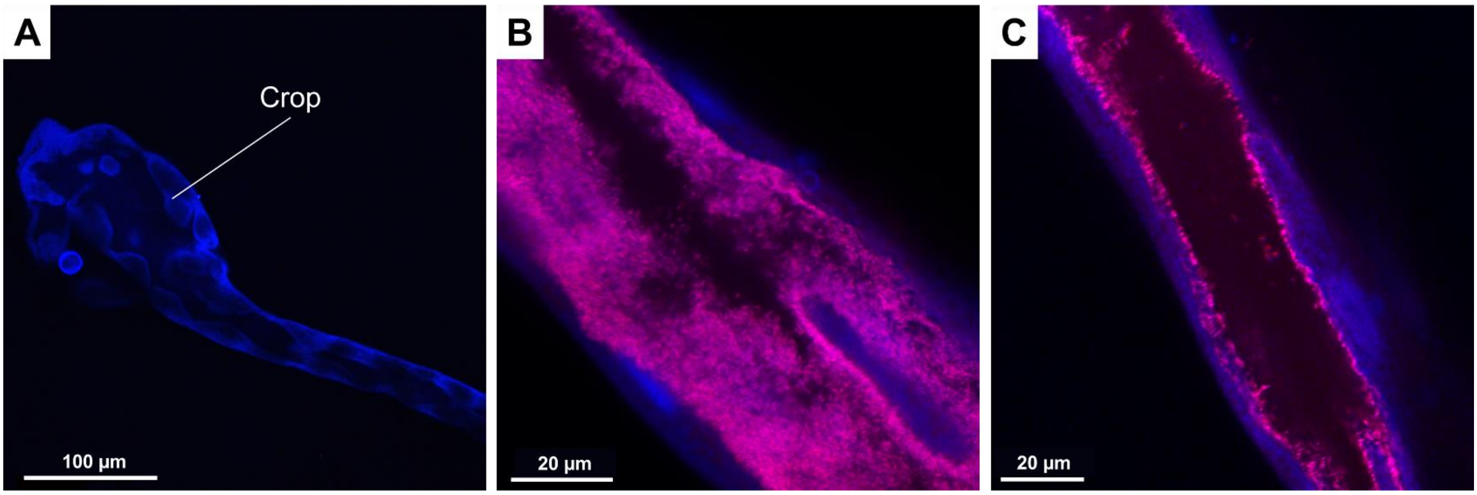

Supplement: Supplemental file 1 — Supplemental material. Download spectrum.00457-22-s0001.pdf, PDF file, 0.4 MB [file spectrum.00457-22-s0001.pdf]
